# Diflunisal‐loaded poly(propylene sulfide) nanoparticles decrease *S. aureus*‐mediated bone destruction during osteomyelitis

**DOI:** 10.1002/jor.24948

**Published:** 2020-12-20

**Authors:** Caleb A. Ford, Thomas J. Spoonmore, Mukesh K. Gupta, Craig L. Duvall, Scott A. Guelcher, James E. Cassat

**Affiliations:** ^1^ Department of Biomedical Engineering Vanderbilt University Nashville Tennessee USA; ^2^ Department of Chemical and Biomolecular Engineering Vanderbilt University Nashville Tennessee USA; ^3^ Department of Medicine Vanderbilt University Medical Center Nashville Tennessee USA; ^4^ Vanderbilt Center for Bone Biology Vanderbilt University Medical Center Nashville Tennessee USA; ^5^ Department of Pediatrics Vanderbilt University Medical Center Nashville Tennessee USA; ^6^ Department of Pathology, Microbiology, and Immunology Vanderbilt University Medical Center Nashville Tennessee USA; ^7^ Vanderbilt Institute for Infection, Immunology, and Inflammation Vanderbilt University Medical Center Nashville Tennessee USA

**Keywords:** antivirulence, drug delivery, nanoparticle, osteomyelitis, *Staphylococcus aureus*

## Abstract

Osteomyelitis is a debilitating infection of bone that results in substantial morbidity. *Staphylococcus aureus* is the most commonly isolated pathogen causing bone infections and features an arsenal of virulence factors that contribute to bone destruction and counteract immune responses. We previously demonstrated that diflunisal, a nonsteroidal anti‐inflammatory drug, decreases *S. aureus*‐induced bone destruction during osteomyelitis when delivered locally from a resorbable drug delivery depot. However, local diflunisal therapy was complicated by bacterial colonization of the depot's surface, highlighting a common pitfall of devices for local drug delivery to infected tissue. It is, therefore, critical to develop an alternative drug delivery method for diflunisal to successfully repurpose this drug as an antivirulence therapy for osteomyelitis. We hypothesized that a nanoparticle‐based parenteral delivery strategy would provide a method for delivering diflunisal to infected tissue while circumventing the complications associated with local delivery. In this study, we demonstrate that poly(propylene sulfide) (PPS) nanoparticles accumulate at the infectious focus in a murine model of staphylococcal osteomyelitis and are capable of efficaciously delivering diflunisal to infected bone. Moreover, diflunisal‐loaded PPS nanoparticles effectively decrease *S. aureus*‐mediated bone destruction, establishing the feasibility of systemic delivery of an antivirulence compound to mitigate bone pathology during osteomyelitis.

## 1 INTRODUCTION

Osteomyelitis, or inflammation of bone, is commonly caused by bacterial infection. This disease afflicts an estimated 1 in 4000 people annually and is projected to impact up to 30% of orthopedic procedures.^1^, ^2^ Due in part to the widespread emergence of antimicrobial resistance, treatment of osteomyelitis can be extremely difficult.^3^, ^4^ Efforts to cure osteomyelitis often involve invasive debridement procedures and long‐term antibiotic therapy that together result in substantial strain on the patient and healthcare system.^2^, ^5^, ^6^, ^7^ *Staphylococcus aureus*, a Gram‐positive bacterium, is the most common etiologic agent of osteomyelitis.^1^ *S. aureus* possesses an arsenal of virulence factors that lyse host cells, including skeletal cells, thereby contributing to osteomyelitis‐induced bone loss.^8^ Thus, effective therapies are necessary to ameliorate concomitant morbidities such as bone loss that may increase the risk of fracture or treatment failure.

Antivirulence therapies inhibit bacterial virulence pathways without directly impacting bacterial viability and are actively being investigated as adjunctive treatment strategies.^3^ We have recently demonstrated the antivirulence potential of diflunisal, a nonsteroidal anti‐inflammatory drug, to decrease *S. aureus*‐induced bone destruction in a murine osteomyelitis model.^9^ Diflunisal inhibits the quorum‐sensing *agr* pathway of *S. aureus*, limiting production of numerous virulence factors including cytolytic toxins.^10^ In previous studies, local delivery of diflunisal from resorbable poly(ester urethane) foams significantly reduced bone resorption.^9^, ^11^ While local delivery presents the advantage of achieving high drug concentrations near target sites, the avascular delivery depot can function as a nidus for bacterial colonization.^11^, ^12^, ^13^ Thus, effective delivery of diflunisal and other antivirulence compounds requires an alternative method to avoid exacerbation of infection.

While parenteral therapy potentially circumvents the challenges of local delivery devices, diflunisal is hydrophobic and therefore has low aqueous solubility. Encapsulation of compounds within nanoparticles has enabled effective systemic delivery of hydrophobic drugs and demonstrated distribution to target sites.^14^, ^15^, ^16^, ^17^, ^18^ Our group has previously shown that poly(propylene sulfide) (PPS) nanoparticles provide a reactive oxygen species (ROS)‐responsive carrier for delivery of the Gli2 inhibitor, GANT58, to sites of bone cancer metastases.^18^ The PPS nanoparticles distributed preferentially to tumor‐bearing limbs compared to contralateral limbs, presumably due to increased vascular permeability at tumor sites that allows for nanoparticle extravasation and decreased lymphatic drainage. These phenomena allow for nanoparticle retention and are known as the enhanced permeability and retention (EPR) effect.^19^ Furthermore, PPS‐based biomaterials break down in the presence of high levels of ROS,^20^, ^21^, ^22^ providing a potential mechanism for targeted drug release at inflamed sites. However, few studies have investigated systemically (e.g., intravenously) delivered nanoparticles in the context of osteomyelitis.^23^, ^24^, ^25^, ^26^

The objectives of this study were to understand the biodistribution of PPS nanoparticles during osteomyelitis and evaluate the efficacy of diflunisal‐loaded nanoparticles in limiting *S. aureus*‐induced bone loss. We hypothesized that PPS nanoparticles would accumulate at infectious foci during osteomyelitis and that diflunisal‐loaded PPS nanoparticles would limit *S. aureus*‐mediated cortical bone destruction. To test these hypotheses, we evaluated PPS nanoparticle delivery in a murine model of osteomyelitis and investigated the efficacy of diflunisal‐loaded PPS nanoparticles both in vitro and in vivo.

## 2 MATERIALS AND METHODS

### 2.1 Cell lines, bacterial strains, and reagents

The murine preosteoblast MC3T3‐E1 subclone 4 cell line was obtained from the American Type Culture Collection. The cells were propagated in a humidified 37°C incubator with 5% CO_2_ and maintained in ⍺‐MEM (Gibco #A1049001; Thermo Fisher Scientific) supplemented with 10% fetal bovine serum (Bio‐Techne) and 1X penicillin–streptomycin (Thermo Fisher Scientific). An erythromycin‐sensitive derivative of the methicillin‐resistant *S. aureus* USA300‐lineage strain LAC (AH1263) was used for all experiments as it represents the most commonly isolated clonal complex causing musculoskeletal infection in the United States.^27^, ^28^ For bacterial growth, unless otherwise noted, 5‐ml cultures were grown in tryptic soy broth at 37°C, shaking at 180 rpm. Diflunisal, dimethylformamide (DMF), dioxane, *N*,*N*‐dimethylacrylamide (DMA), propylene sulfide, dimethyl sulfoxide (DMSO), 2,2’‐azobis(isobutyronitrile) (AIBN), Nile red, and hydrogen peroxide (H_2_O_2_) were purchased from MilliporeSigma. Cy7‐amine was purchased from Lumiprobe. DMA was purified by distillation under reduced pressure before polymerization. PPS (10 kDa), poly(propylene sulfide)‐4‐cyano‐4‐(ethylsulfanylthiocarbonyl)sulfanylpentanoic acid (PPS_135_‐ECT), and poly(benzoyloxypropyl methacrylamide) (pHPMA‐Bz) were synthesized as described previously.^29^

### 2.2 Synthesis and characterization of the polymer

The diblock copolymer consists of PPS (135 repeat units) and DMA (149 repeat units). All copolymer solutions were synthesized with 1 repeat unit of pentafluorophenyl acrylate (PFPA) for which Cy7‐amine was substituted to provide a fluorescent marker for in vivo tracking. Synthesis of PPS_135_‐*b*‐p(Cy7_1_‐*ran*‐DMA_149_) was conducted as previously published^18^ with modifications as follows. Reversible addition–fragmentation chain‐transfer (RAFT) polymerization of the second block (i.e., the DMA block with single repeat unit of PFPA) of PPS_135_‐*b*‐p(PFPA_1_‐*ran*‐DMA_149_) was performed with a 5‐to‐1 molar ratio of PPS_135_‐ECT (macro chain transfer agent, macroCTA) to initiator (AIBN). The polymerization was conducted in a 10‐ml, round‐bottom reaction vessel containing 0.02687 mmol (268.7 mg) macroCTA, 4.02 mmol (415 μl) DMA, 0.0067 mmol (1.1 μl) PFPA, 0.0054 mmol (88.3 μl of 10‐mg/ml AIBN dioxane) AIBN, and 4 ml of 1:1::DMF:dioxane solvent. The reaction vessel was purged with nitrogen, and the resulting solution was stirred at 65°C for 24 h, after which time the reaction was quenched at −80°C. To graft Cy7‐amine, 0.5 ml of thawed polymer solution was removed and replaced with 0.5ml of DMSO containing 0.00672 mmol (4.8 mg) of Cy7‐amine for 24 h, stirring at room temperature. The resulting solution was dialyzed against methanol and deionized water for 24 h each before lyophilization. A ^1^H NMR spectrum of polymer was collected in CDCl_3_ with a Brüker 400 MHz spectrometer as before.^30^

### 2.3 Fabrication and characterization of nanoparticles

Following synthesis of the polymer, micellar nanoparticles were fabricated using an oil‐in‐water emulsion technique. Nanoparticles were fabricated using two techniques: bulk solvent evaporation for small batches to optimize drug loading parameters and a microfluidics approach to scale up nanoparticle production for animal experiments. For diflunisal loading experiments, batches of nanoparticle solutions were fabricated using a bulk solvent evaporation procedure performed previously.^18^ Briefly, PPS_135_‐*b*‐p(Cy7_1_‐*ran*‐DMA_149_) (10.0 mg) was codissolved with diflunisal (1.0 mg) in chloroform (0.1 ml) and added dropwise to a vial containing vigorously stirring phosphate‐buffered saline (PBS). In addition, pHPMA‐Bz was added to a subset of batches at a ratio of 1:1 pHPMA‐Bz:diflunisal by mass to determine the influence of facilitated π–π stacking on diflunisal encapsulation as shown before.^31^ The chloroform‐PBS biphasic solution was left stirring overnight to allow chloroform evaporation and micelle formation. The resulting micelle solution was passed through a 0.45‐μm syringe filter. Diflunisal loading was quantified by the aqueous concentration calculated from the measurement of diflunisal fluorescence (Ex. 310 nm, Em. 420 nm) with reference to a standard curve using a microplate reader (Tecan Infinite 500; Tecan Group Ltd.). To characterize ROS‐mediated release of loaded agents, Nile red release from nanoparticles was measured as previously reported at the stated H_2_O_2_ concentrations.^18^, ^21^, ^32^

After determining optimal parameters for drug loading, nanoparticles were fabricated in large batches by microfluidics processes as described previously for animal studies.^33^ Briefly, PPS_135_‐*b*‐p(Cy7_1_‐*ran*‐DMA_149_) (60.0 mg) was codissolved with pHPMA‐Bz (6.0 mg) and/or diflunisal (6.0 mg) in methanol (0.6 ml) and mixed with sterile PBS using a benchtop NanoAssemblr (Precision Nanosystems, Inc.). All formulations were prepared with a 10:1::aqueous:organic flow rate ratio and 4 ml/min total flow rate. Methanol was removed using a rotovap heated to 40°C for 30 min. Resulting solutions were passed through 0.45‐μm syringe filters. All nanoparticles contained Cy7‐grafted polymer for imaging purposes. Dif‐NPs refers to nanoparticles loaded with diflunisal and pHPMA‐Bz. Blank‐NPs refers to blank nanoparticles containing pHPMA‐Bz only and serves as the vehicle control for Dif‐NPs. Empty‐NPs refers to empty nanoparticles and are used to visualize biodistribution of the nanoparticle. Dynamic light scattering (DLS) was used to measure the hydrodynamic diameter of synthesized nanoparticles in PBS using a Malvern Zetasizer Nano‐ZS (Malvern Instruments Ltd.) equipped with a 4 mW He–Ne laser operating at *λ* = 632.8 nm.

### 2.4 Biodistribution of PPS nanoparticles

Empty‐NPs were delivered to 7–8‐week‐old female FVB/NJ mice (*n* = 4 mice) by tail vein injection. Mice were imaged at 1 h (under 1%–5% isoflurane anesthesia) and 24 h (immediately post‐euthanasia) following nanoparticle‐injection using an IVIS Spectrum imaging system (PerkinElmer). Cy7 detection (Ex: 675 nm, Em: 780 nm) was used to characterize nanoparticle distribution in whole‐body images with a 5‐s fluorescent exposure on high intensity and small binning with an f/stop value of 8. Images were analyzed using ROI analysis with Living Image Software.

### 2.5 Preparation of concentrated supernatants

One colony of *S. aureus* from a tryptic soy agar plate was used to inoculate a 15‐ml sample of Roswell Park Memorial Institute (RPMI; Corning) supplemented with 10 g/L casamino acids (MilliporeSigma) in a 50‐ml conical tube. Samples were supplemented with either 15‐µl DMSO, 10‐µg/ml diflunisal (solubilized in 15‐μl DMSO), Blank‐NPs, or Dif‐NPs (at a final concentration of 10‐μg/ml diflunisal). Samples were prepared in triplicate. Bacteria were grown for 15 h at 37°C and 180 rpm. The triplicate cultures of each group were combined into a single culture of approximately 45‐ml volume and concentrated in Amicon Ultra 3‐kDa nominal molecular weight columns as done previously.^9^, ^11^ Resulting samples were filter‐sterilized and frozen at −80°C.

To measure the effect of diflunisal and nanoparticles on bacterial growth, 15‐ml cultures were supplemented with DMSO, 10μg/ml diflunisal, PBS, or Blank‐NPs. The bacterial cultures were subsequently grown in 200‐μl volumes in round‐bottom, tissue culture‐treated 96‐well plates for 15 h at 37°C. The optical density at 600 nm (OD_600_) was recorded each hour to monitor bacterial growth using a BioTek Synergy HT microplate reader (BioTek Instruments, Inc.). The initial OD_600_ reading was subtracted from each well to serve as a baseline.

### 2.6 MC3T3 cytotoxicity assay

MC3T3 cytotoxicity was analyzed as reported previously.^9^, ^11^ Cells were intoxicated 12–24 h after initial seeding in 96‐well plates with either prepared supernatants or vehicle (RPMI containing casamino acids) at 20% vol/vol for 22 h. Cell viability was determined using CellTiter 96® AQueous One Solution (Promega) according to manufacturer's instructions. The percent viability following treatment was expressed as a percentage of the absorbance of the vehicle‐treated wells.

### 2.7 Murine model of osteomyelitis

This study was approved by the Institutional Animal Care and Use Committee of Vanderbilt University Medical Center and conducted in compliance with Animal Welfare Regulations and the principles of the Guide for the Care and Use of Laboratory Animals. All procedures were performed in an ABSL‐2 facility. Following 1 week of acclimation, osteomyelitis was induced in 7–8‐week‐old female C57BL/6J, FVB/NJ, or BALB/cJ mice (Jackson Laboratory) as previously described with the difference that buprenorphine (analgesic) was administered as a long‐acting dose (Buprenorphine‐SR; ZooPharm).^8^ An inoculum of 10^6^ colony‐forming units in 2‐μl PBS was delivered into femurs. Mice that experienced more than 20% weight loss following infection (humane endpoint determined a priori in consultation with veterinary staff) were euthanized and excluded from analyses. Dif‐NPs and Blank‐NPs (*n* = 12) were injected via tail vein daily at a volume of 100 μl starting approximately 1 h postinfection. *N* = 12 was based on power calculations from the initial pilot study (*n* = 5) analyzing cortical bone loss between these groups and is the primary comparison of the study. These treatments were performed in an unblinded manner and using a random group assignment by cage. The Dif‐NP group received the nanoparticle treatment immediately before the Blank‐NP group. A PBS injection was used as a control (*n* = 5) for comparison. Mice were euthanized at multiple time points up to 14 days postinfection and imaged by IVIS as above. The infected femur, contralateral femur, liver, kidneys, and spleen were then removed and imaged ex vivo by IVIS. To account for intrinsic autofluorescence of tissues, the fluorescence intensity of all ex vivo organs was normalized to the fluorescence intensity of the respective organs harvested from a PBS‐injected control mouse at each time point. Following IVIS imaging, infected femurs were then analyzed by μCT as described previously.^8^ Briefly, axial images of each femur were captured with 5.0‐μm voxels at 70 kV, 200 μA, 2000 projections per rotation, and an integration time of 350 ms in a 10.24 mm field‐of‐view. Each imaging scan comprised 1635 slices (8.125 mm), centered on the mid‐diaphysis near the inoculation site. Volume of interest was limited to the original cortical bone, and any destruction was selected by drawing contours on the endosteal and periosteal surfaces. A subset (*n* = 5) of Blank‐NP and Dif‐NP femurs were decalcified, paraffin‐embedded, and stained by a modified hematoxylin and eosin stain as reported previously.^34^

To understand the effect of systemically delivering a nanoparticle‐free diflunisal formulation, mice were treated with a saturated solution of free diflunisal in PBS (*n* = 16) and compared to mice receiving Dif‐NP injections in studies adding additional mice (*n* = 9) to the Dif‐NP treatment group. Within these studies an additional PBS control (*n* = 4) served as a baseline. Across three experimental trials analyzing cortical bone destruction group sizes were as follows: PBS (*n* = 9), Blank‐NP (*n* = 12), Dif‐NP (*n* = 21), and free‐drug diflunisal (*n* = 16). The free‐drug diflunisal solution was prepared by mixing twice the maximum aqueous solubility of diflunisal (maximum solubility: 14.5 μg/ml) in PBS, heating in a 37°C water bath for 30 min, and vortexing for 30 min. The solution was passed through a 0.45‐μm syringe filter before treatment. In a separate experiment to further determine the impact of Dif‐NPs on bacterial burdens in vivo, an additional group of mice (*n* = 5) were treated and euthanized at Day 7, and bacterial burdens were assessed as conducted previously.^34^ In total, 86 animals were used to complete these studies.

### 2.8 Statistical evaluation

Differences in diflunisal encapsulation, Nile red release, supernatant‐mediated cytotoxicity, and OD_600_ growth curves were assessed by two‐way analysis of variance (ANOVA). Differences in nanoparticle biodistribution were assessed by paired Student's *t*‐test. Differences in fluorescence intensity of organs were assessed by one‐way ANOVA or two‐way ANOVA as stated. Differences in cortical bone destruction and bacterial burdens were compared using a one‐way ANOVA. A *p* value of .05 was considered significant for all analyses. All statistical analyses were performed with GraphPad Prism.

## 3 RESULTS

### 3.1 PPS diblock copolymer nanoparticle synthesis and cargo release

**Figure 1:**
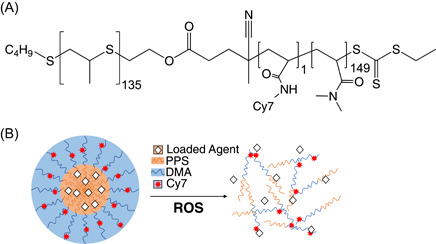
PPS_135_‐*b*‐p(Cy7_1_‐*ran*‐DMA_149_) forms reactive oxygen species (ROS)‐responsive nanoparticles. (A) PPS_135_‐*b*‐p(Cy7_1_‐*ran*‐DMA_149_) structure contains repeat units of propylene sulfide (orange), Cy7 (red), and *N*,*N*‐dimethylacrylamide (DMA; blue). (B) Schematic of micellar poly(propylene sulfide) (PPS) nanoparticles encapsulating a loaded agent. Upon oxidation by ROS, PPS nanoparticles become unstable due to PPS conversion from hydrophobic to hydrophilic, releasing the loaded agent

To generate the building blocks necessary for fluorescent nanoparticle synthesis, PPS‐*b*‐p(Cy7_1_‐*ran*‐DMA_149_) polymer (Figure 1A) was synthesized by RAFT polymerization. Polymer structure was confirmed by ^1^H NMR (Figure S1). An oil‐in‐water emulsion formed the micellar nanoparticles in which the hydrophilic DMA blocks compose the hydrophilic corona and the hydrophobic PPS blocks compose the ROS‐responsive core which releases loaded drug upon destabilization (Figure 1B).

### 3.2 Formation of diflunisal‐loaded PPS nanoparticles for drug delivery

**Figure 2:**
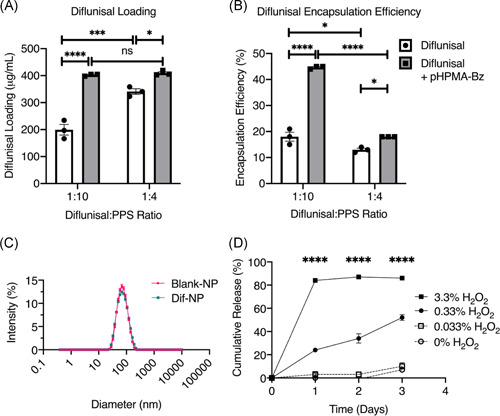
Poly(propylene sulfide) (PPS) nanoparticles effectively load diflunisal with no effect on nanoparticle size. Diflunisal encapsulation by bulk solvent evaporation was quantified by (A) loading and (B) encapsulation efficiency. For both drug‐to‐polymer ratios, the excipient pHPMA‐Bz was tested (gray bars). *N* = 3. Error bars represent mean ± *SEM*. *p < .05, ***p < .001, ****p < .0001, and ns denotes no significance (*p* > .05) as determined by two‐way analysis of variance (ANOVA). (C) Nanoparticle hydrodynamic diameter was analyzed by dynamic light scattering for Blank‐NPs and Dif‐NPs. (D) Cumulative release measured as the loss of fluorescence of Nile red (a dye that is fluorescent in hydrophobic environments such as the PPS core) from PPS nanoparticles exposed to various concentrations of the reactive oxygen species H_2_O_2_. Error bars represent mean ± *SEM*. ****p < .0001 between the individual 0.33% and 3.3% H_2_O_2_ groups and all other groups at the given time point as determined by two‐way ANOVA

To determine the optimal process for encapsulation of diflunisal within PPS nanoparticles (Dif‐NPs), the quantity of loaded drug and encapsulation efficiency of two different drug‐to‐polymer ratios were characterized. The addition of pHPMA‐Bz as an excipient was also tested to determine the benefits of facilitated π–π stacking on diflunisal encapsulation. Increasing the drug‐to‐polymer ratio from 1:10 to 1:4 was found to improve drug loading (Figure 2A); however, the encapsulation efficiency was substantially greater for the 1:10 formulation (Figure 2B). Use of pHPMA‐Bz approximately doubled diflunisal loading in a 1:10 formulation compared to PPS nanoparticles without pHPMA‐Bz (Figure 2A), resulting in the formulation with the highest weight percentage of diflunisal. Thus, the optimal Dif‐NP formulation was determined to be a drug‐to‐polymer ratio of 1:10 with addition of pHPMA‐Bz in a 1:1 mass ratio with diflunisal. To examine the influence of diflunisal loading on nanoparticle size, Dif‐NPs and Blank‐NPs were evaluated by DLS to determine the average hydrodynamic diameter (Figure 2C). The observed diameters for Blank‐NPs and Dif‐NPs were 65.4 ± 0.4 and 65.4 ± 0.4 nm, respectively, showing no change upon drug loading. Similarly, the polydispersity indices for Blank‐NPs and Dif‐NPs were 0.138 ± 0.004 and 0.163 ± 0.009, respectively, demonstrating a comparable dispersity of nanoparticle size within each formulation. Therefore, a 1:10 drug‐to‐polymer ratio coencapsulated with pHPMA‐Bz was chosen as the optimal formulation for diflunisal loading in PPS nanoparticles. Finally, to confirm ROS potentiates drug release from PPS nanoparticles, Nile red‐loaded nanoparticles were treated with H_2_O_2_ and loss of fluorescence was measured as a sign of drug release (Figure 2D).

### 3.3 Systemically administered nanoparticles accumulate at infected femurs

**Figure 3:**
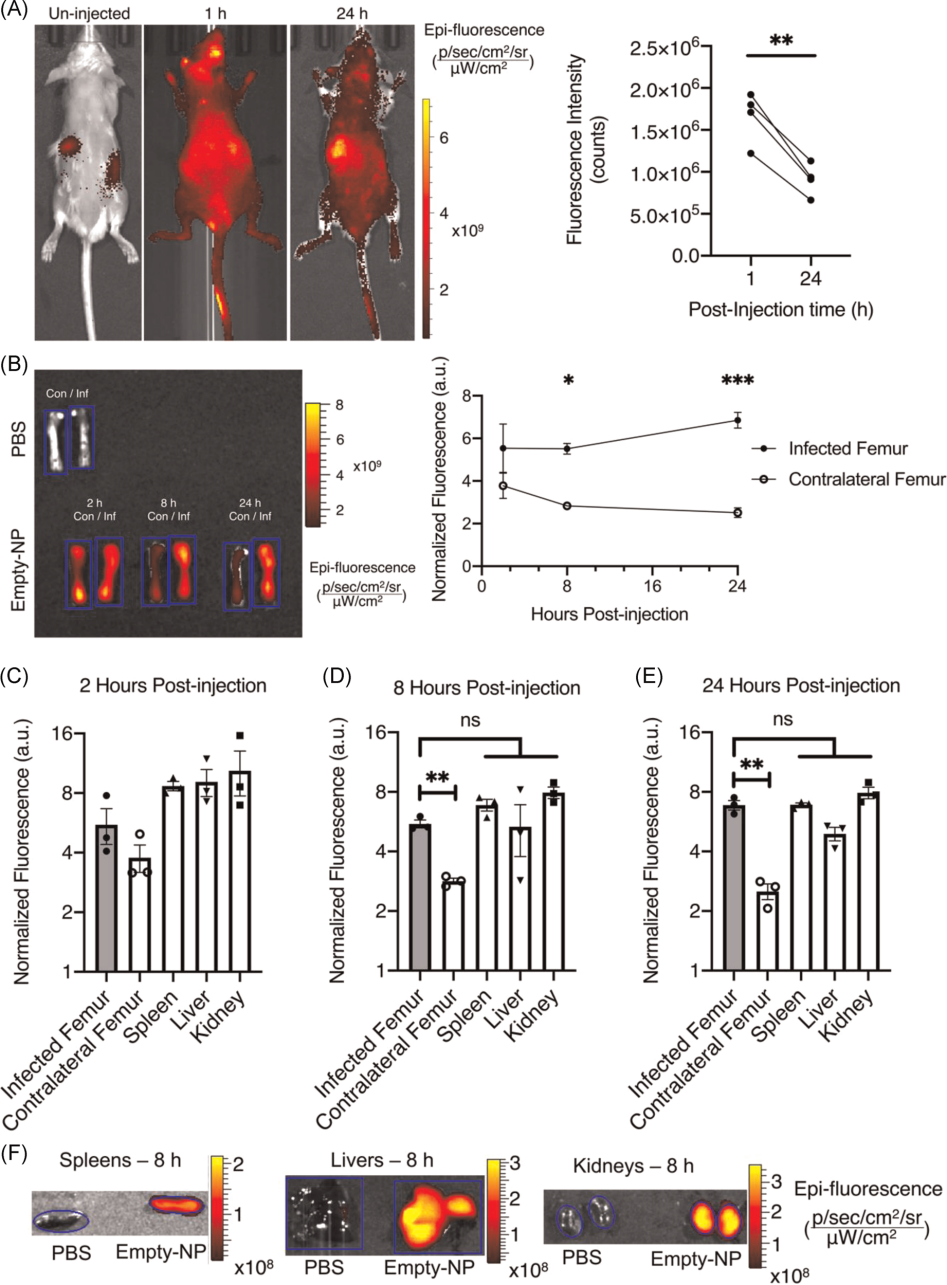
Poly(propylene sulfide) (PPS) nanoparticles accumulate at infected femurs. (A) Whole‐body IVIS images of Cy7 in uninfected mice at 1 and 24 h following injection with Empty‐NPs 1‐ and 24 h postinjection. ROI analysis of the entire animal was quantified at 1‐ and 24 h postinjection (*n* = 4). **p < .01 as determined by paired Student's *t*‐test. (B) A representative image with corresponding numerical analysis of Cy7 fluorescent signal in infected (Inf) and contralateral (Con) femurs following injection of Empty‐NPs in infected mice at 2‐, 8‐, and 24 h postinjection. Blue outlines represent ROIs used for numerical analyses. A set of femurs from a phosphate‐buffered saline (PBS)‐injected animal is shown as the nonfluorescent control to which the fluorescence intensity was normalized. *N* = 3 mice per group. Error bars represent mean ± *SEM*. *p < .05 as determined by two‐way analysis of variance (ANOVA). At 2 h (C), 8 h (D), and 24 h (E), organs (spleen, liver, and kidneys) were dissected and similarly analyzed to determine difference from fluorescent intensity of the femurs and organs of the same mice in (B). As before, *n* = 3 mice per group. Error bars represent mean ± *SEM*. **p < .01 as determined by one‐way ANOVA. (F) A representative image at 8 h postinjection is shown for each organ from an Empty‐NP‐injected mouse relative to that of the PBS‐injected control mouse to demonstrate the ROIs used for the analyses

Having identified an optimal nanoparticle formulation, we sought to determine the systemic biodistribution of PPS nanoparticles to infectious sites in vivo. To first understand the biodistribution in healthy animals, uninfected FVB/NJ mice were injected with Empty‐NPs via the lateral tail vein and imaged in the IVIS system at 1 and 24 h postinjection for Cy7 fluorescence. As expected, uninjected animals showed autofluorescence in the gastrointestinal tract from regular chow.^35^ Measurements of Cy7 fluorescence demonstrated that Empty‐NPs distributed systemically throughout the mouse within 1 h postinjection, consistent with intravenous administration (Figure 3A). The Cy7 signal persisted after 24 h at a decreased intensity, suggesting that nanoparticles were still present at lower concentrations (Figure 3A). Next, FVB/NJ mice were subjected to osteomyelitis and injected with Empty‐NPs 24 h postinfection to assess the biodistribution of nanoparticles following infection. Previous experiments in our group have predominantly used C57BL/6J mice to model osteomyelitis; however, we sought a nonpigmented mouse for imaging and confirmed that bacterial burdens on postinfection Day 7 did not differ between C57BL/6J, BALB/cJ, and FVB/NJ mice (Figure S2). Organs were dissected and immediately assessed by IVIS to determine Cy7 fluorescent signal intensity at 2‐, 8‐, and 24 h postinjection. The infected femurs showed accumulation compared to the contralateral femurs over 24 h (Figure 3B). Nanoparticle accumulation was also compared to three highly vascularized organs (livers, kidneys, and spleens) up to 24 h postinjection (Figure 3C–F). At all tested time points, the well‐vascularized organs displayed consistently high Cy7 signal intensity. These data establish that a single administration of Empty‐NPs yields greater accumulation at the infected femur relative to the contralateral femur 24 h postinjection.

### 3.4 Dif‐NPs limit *S. aureus* cytotoxicity

**Figure 4:**
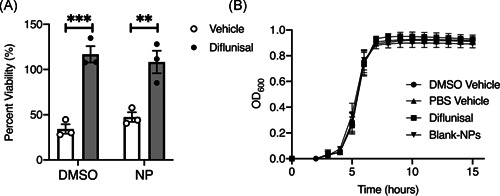
Dif‐NPs inhibit *Staphylococcus aureus* cytotoxicity toward MC3T3s. (A) MC3T3 murine preosteoblast cells were intoxicated with 20% (vol/vol) of concentrated supernatant from *S. aureus* grown in the presence of vehicle control (dimethyl sulfoxide [DMSO]), nanoparticle vehicle control (Blank‐NP), diflunisal (10 µg/ml in DMSO), or diflunisal‐loaded nanoparticles (10 µg/ml encapsulated in poly(propylene sulfide) nanoparticles). MC3T3 viability is depicted as a percentage relative to mock intoxication with sterile Roswell Park Memorial Institute. *N* = 3 independent replicates. Error bars represent mean ± *SEM*. **p < .01 and ***p < .001 as determined by two‐way analysis of variance. (B) Optical density of *S. aureus* grown in presence of vehicle controls (phosphate‐buffered saline [PBS] and DMSO), diflunisal (10 µg/ml in DMSO), or Blank‐NPs. *N* = 3 independent replicates. Error bars represent mean ± *SEM*

To determine the antivirulence efficacy of Dif‐NPs, we utilized a previously published in vitro method to assess the influence of Dif‐NPs on staphylococcal cytotoxicity towards a preosteoblast cell line.^9^ MC3T3 cells were exposed to concentrated *S. aureus* supernatants prepared from bacterial cultures treated with Blank‐NPs, Dif‐NPs, or diflunisal as a free drug. Diflunisal, delivered either as free drug or encapsulated within PPS nanoparticles, significantly inhibited the cytotoxicity of *S. aureus* supernatants (Figure 4A). To determine the effects of diflunisal and PPS nanoparticles on bacterial growth, optical density of *S. aureus* cultures grown in the presence of 10‐µg/ml diflunisal or Blank‐NPs was assessed over a 15 h time period. Bacterial growth was also assessed in the presence of DMSO and PBS as vehicle controls for free‐drug diflunisal and Blank‐NPs, respectively. We did not observe a significant difference in OD_600_ between any of the groups (Figure 4B), suggesting that neither component of Dif‐NPs (diflunisal or Blank‐NPs) hinders bacterial growth. Thus, diflunisal released from Dif‐NPs inhibits *S. aureus* cytotoxicity, and neither component of Dif‐NPs affects bacterial growth.

### 3.5 Dif‐NPs decrease *S. aureus*‐induced cortical bone loss during osteomyelitis

**Figure 5:**
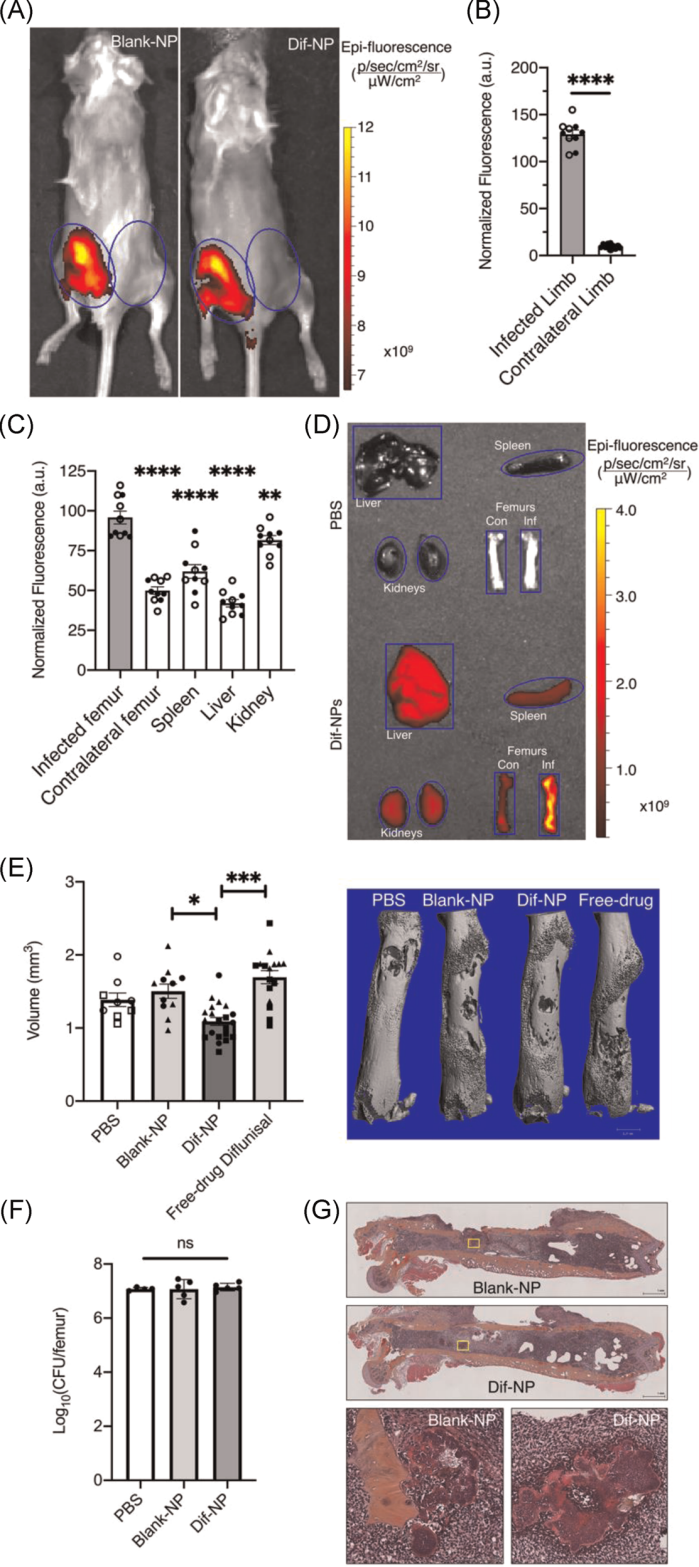
Dif‐NPs decrease *Staphylococcus aureus*‐induced bone destruction during osteomyelitis. (A) Representative IVIS images of mice 14 days postinfection following daily tail vein injections of either Dif‐NPs (*n* = 5) or Blank‐NPs (*n* = 5). Blue circles denote ROIs for quantitative analyses. (B) Fluorescence of infected and contralateral limbs in both groups of mice were assessed using ROI analysis of the limbs. Filled circles represent mice treated with Blank‐NPs, and open circles represent mice treated with Dif‐NPs. Fluorescence intensity was normalized to the intensity of the corresponding ROI of phosphate‐buffered saline (PBS)‐injected animals. Error bars represent mean ± *SEM*. ****p < .0001 as determined by Student's *t*‐test. (C) Quantification of dissected organs ex vivo 14 days postinfection following daily tail vein injections of Dif‐NPs or Blank‐NPs (same groups of mice as in A). Filled circles represent organs of mice treated with Blank‐NPs, and open circles represent organs of mice treated with Dif‐NPs. Error bars represent mean ± *SEM*. **p < .01 and ****p < .0001 as determined by one‐way analysis of variance (ANOVA). (D) A representative image of the analyzed dissected organs of 5C is shown: livers, spleens, kidneys, infected (Inf) femurs, and contralateral (Con) femurs. Organs of a PBS‐injected mouse and a Dif‐NP‐injected mouse are shown with ROIs. The organs of the PBS‐injected animal do not show fluorescence intensity above the image threshold. (E) Quantification of cortical bone destruction 14 days postinfection with *S. aureus* and following daily treatment with PBS, Blank‐NPs, Dif‐NPs, or free‐drug diflunisal via tail vein injection. *N* = 9–21 mice per group. One mouse in the Blank‐NPs group experienced more than 20% weight loss and was euthanized. Different symbols (circles, triangles, and squares) represent three independent trials that included the groups as indicated by the corresponding symbols. Effect size (Hedges’ g) between Blank‐NP and Dif‐NP groups = −1.500 (95% confidence interval: −0.684, −2.317). The median femur from each group is shown in a three‐dimensional reconstruction to the right of the graph. Error bars represent mean ± *SEM*. **p < .01 and ****p < .0001 as determined by one‐way ANOVA. (F) Quantification of bacterial burden by colony‐forming units enumeration 7 days postinfection following daily treatment with PBS, Blank‐NPs, or Dif‐NPs. *N* = 5 mice per group. One mouse in the PBS group was euthanized following an adverse response to anesthesia. Error bars represent mean ± *SEM*. ns denotes no significance as determined by one‐way ANOVA. (G) Representative histology images of femurs harvested from mice treated with Blank‐NPs or Dif‐NPs and stained with a modified hematoxylin and eosin stain. Scale bars are as shown in the lower right corner of images

Given the observations that Dif‐NPs inhibit the cytotoxicity of *S. aureus* in vitro and that nanoparticles accumulate at infectious foci, we sought to investigate the therapeutic capability of Dif‐NPs. First, to characterize the distribution of diflunisal‐loaded nanoparticles, femurs and organs of mice injected daily with Dif‐NPs or Blank‐NPs for 14 days were assessed by fluorescent imaging. On Day 14 postinfection, mice treated with either Dif‐NPs or Blank‐NPs were both found to have significant increases in fluorescence intensity in the infected limb compared to the contralateral limb (Figure 5A,B). When assessed ex vivo postdissection, infected femurs at Day 14 postinfection showed significantly greater signal intensity compared to the intensities of all other tested organs (Figure 5C,D). Thus, Dif‐NPs and Blank‐NPs accumulated at the infected femur following 14 days of daily injections.

To determine the ability of Dif‐NPs to decrease *S. aureus*‐induced bone loss during osteomyelitis, infected mice were treated with daily injections of Dif‐NPs or Blank‐NPs starting 1 h postinfection. Infected femurs were isolated at Day 14 and analyzed by microCT to quantify bone loss. Femoral reconstructions upon which calculations were made are shown in Figure S3. Mice treated with Dif‐NPs demonstrated significantly less cortical bone destruction compared to mice treated with Blank‐NPs at Day 14 (Figure 5E). Thus, diflunisal‐loaded PPS nanoparticles decrease *S. aureus*‐induced bone loss in infected femurs. To compare the efficacy of delivering free‐drug (unencapsulated) compared to nanoparticle‐encapsulated diflunisal, infected mice were injected daily with free‐drug diflunisal in PBS or Dif‐NPs. Mice treated with Dif‐NPs demonstrated significantly lower bone destruction than mice treated with free‐drug 14 days postinfection (Figure 5E). Notably, cortical bone loss in PBS‐injected mice did not differ from that of Blank‐NP‐injected mice or free‐drug diflunisal‐injected mice (Figure 5E). Thus, nanoparticle encapsulation resulted in enhanced efficacy of systemically delivered diflunisal, likely as a function of overcoming the limited aqueous solubility of free‐drug diflunisal.

To assess the effect of Dif‐NPs on bacterial burden, a separate cohort of mice were injected with PBS, Dif‐NPs, or Blank‐NPs daily for 7 days postinfection. No differences in bacterial enumeration were measured between the groups (Figure 5F), and histological sections revealed evidence of abscesses in all mice treated with either Blank‐NPs or Dif‐NPs (Figures 5G and S4), suggesting that Dif‐NPs had no effect on bacterial burdens. Thus, diflunisal‐loaded PPS nanoparticles decrease *S. aureus*‐induced bone loss in infected femurs during osteomyelitis without significantly influencing bacterial burdens. Taken together, these data support findings that PPS nanoparticles efficaciously deliver diflunisal to infectious foci to decrease bone destruction during osteomyelitis.

## 4 DISCUSSION

Delivery of hydrophobic drugs such as diflunisal is limited by low aqueous solubility, which can lead to unfavorable pharmacokinetic profiles and poor biodistribution when delivered parenterally.^36^, ^37^ Local delivery systems have been designed to overcome solubility limitations; however, foreign devices are known to be a nidus for bacterial colonization and biofilm formation.^11^, ^12^, ^13^ While some compounds (including diflunisal) can achieve systemic delivery through oral delivery, oral administration is not feasible in all clinical settings (e.g., moribund or perioperative patients), and alternative parenteral options may be advantageous. For such compounds without parenteral compatibility, nanoparticle delivery systems offer a parenteral delivery vehicle for pharmaceuticals to target sites. Although nanoparticle accumulation has not been extensively studied in the context of osteomyelitis, effective treatment of bone infection with systemically administered nanoparticles has been reported.^23^, ^26^ Delivery of antimicrobial compounds using locally administered nanoparticles has also been investigated both in vitro^14^, ^38^ and in vivo,^39^, ^40^, ^41^ but systemic delivery of nanoparticles capable of carrying hydrophobic drugs is under‐investigated in osteomyelitis. Delivery of diflunisal using nanoparticles may provide effective therapy and limit potential complications associated with avascular local delivery devices.

In this study, we evaluated the efficacy of PPS nanoparticles to deliver diflunisal, which we previously demonstrated inhibits *S. aureus*‐induced cortical bone destruction when delivered locally from resorbable poly(ester urethane) scaffolds.^9^, ^11^ We hypothesized that diflunisal‐loaded PPS nanoparticles would accumulate at infectious foci during osteomyelitis and inhibit *S. aureus*‐mediated bone destruction. Reconciling this hypothesis in a model of invasive *S. aureus* disease is crucial given the known limitations of avascular drug depots. Our results indicate that PPS nanoparticles accumulate at infected femurs in a murine model of posttraumatic osteomyelitis. Moreover, we discovered that diflunisal‐loaded PPS nanoparticles effectively mitigated osteomyelitis‐induced bone destruction. Importantly, we also determined that bacterial burdens were unaffected by nanoparticle presence compared with mice treated with PBS alone. Therefore, PPS nanoparticles provide efficacious treatment of diflunisal without exacerbating the infection. Given that PPS nanoparticles accumulate at the site of infection, investigation into other drug cargoes such as novel antivirulence compounds or antimicrobials should be performed. Further research into combined diflunisal‐loaded PPS nanoparticle delivery with a systemically delivered antibiotic is also important given the clinical relevance of administering antivirulence compounds as adjunctive therapies. Future investigations should explore the efficacy of delayed treatment and the optimal timing between administrations of diflunisal‐loaded PPS nanoparticles.

Compared to free‐drug administration via intravenous or oral delivery routes, synthetic nanoparticles offer the potential to accumulate and release loaded compounds at target sites.^14^, ^15^, ^16^, ^17^, ^18^ As described by the EPR effect, both tumors and inflammation result in enhanced vascular permeability allowing for extravasation of nanoparticles.^42^ Our results suggest that PPS nanoparticles accumulate at the infectious foci; however, the exact mechanisms that drive nanoparticle retention during posttraumatic osteomyelitis must be investigated further. One possible mechanism may include phagocytic cell uptake as described in the “ELVIS” effect (extravasation via leaky vasculature followed by inflammatory cell sequestration).^43^, ^44^ Nevertheless, modifications to the nanoparticle chemistry have shown enhanced retention at target sites and allow for further improvement of nanoparticle accumulation in bone in other disease models.^29^ Considering that sites of inflammation and infection are known to produce ROS^45^ and that release of compounds from PPS nanoparticles is responsive to ROS concentration, it is likely that ROS levels at infected sites contribute to drug cargo release within bone. However, more extensive in vivo analyses must be performed to conclude that ROS‐mediated degradation is the primary mechanism of drug release at the infectious site.

Limitations of this study include the use of only one bacterial strain and a single infection model. Due to the different virulence and metabolic profiles of various bacterial pathogens, future studies should explore the use of alternative bacterial species to understand PPS nanoparticle delivery to different infectious foci. *S. aureus* was chosen to model infection in bone due to its high association with bone infection, but PPS nanoparticles delivered to infections in other infected organs have yet to be studied. Similarly, a focus on treatment times greater than 14 days should be investigated to understand the extent to which longer treatments may impact therapeutic outcomes. In the future, experiments should test the ability of PPS nanoparticles to decrease deleterious side effects of toxic agents that would otherwise limit systemic delivery of the drug. Nevertheless, this study suggests PPS nanoparticles efficaciously deliver drug to infectious foci and promotes investigation of nanoparticles as small molecule carriers for osteomyelitis therapy.

## AUTHOR CONTRIBUTIONS

Caleb A. Ford and Thomas J. Spoonmore contributed to study design, data acquisition, data analysis, data interpretation, and manuscript preparation and editing. Mukesh K. Gupta contributed to data acquisition, data analysis, and manuscript editing. Craig L. Duvall contributed to data interpretation, funding acquisition, and manuscript editing. Scott A. Guelcher and James E. Cassat contributed to study design, data interpretation, funding acquisition, and manuscript editing. All authors have read and approved the final submitted manuscript.

## Supporting information

Supporting information.
