# Supplementary material for: Diflunisal‐loaded poly(propylene sulfide) nanoparticles decrease S. aureus‐mediated bone destruction during osteomyelitis
Source: J Orthop Res. 2020 Dec 20;39(2):426–37. doi: 10.1002/jor.24948 (PMC7855846; doi:10.1002/jor.24948)
Supplement: Supplementary file 1 — Supporting information. [file JOR-39-426-s001.docx]

Supplemental Materials

Figure S1. ^1^H NMR spectral analysis of PPS_135_-*b*-p(Cy7_1_-*ran*-DMA_149_) in CDCl_3_. Hydrogen peaks of monomer repeats shown with corresponding labels α through ζ. Tetramethylsilane (TMS) reference and CHCl_3_ contaminant also labeled at expected chemical shifts.

**Figure S2. Bacterial burdens of *S. aureus* in different mouse strains are similar.** Quantification of bacterial burden by CFU enumeration 7 days post-infection in C57Bl/6J, FVB/NJ, and BALB/cJ mice. N=5 mice per group. Error bars represent mean ± SEM. ns denotes no significance (*p*>0.05) as determined by 1-way ANOVA.

**Figure S3. MicroCT three-dimensional reconstructions of infected femurs following diflunisal nanoparticle treatment during osteomyelitis.** μCT reconstructions of femurs subjected to either PBS, Blank-NPs, Dif-NPs, or free-drug diflunisal via tail vein injection. N=9, 11, 21, or 16 mice per group as shown. One mouse in the Blank-NP group experienced >20% weight loss and was euthanized.

**Figure S4. Qualitative images of bacterial abscess formation following diflunisal nanoparticle treatment of osteomyelitis.** Representative histology images of *S. aureus* microcolonies within abscesses of infected femurs harvested from mice treated with Blank-NPs or Dif-NPs and stained with a modified hematoxylin and eosin stain. Each image corresponds to a *S. aureus* microcolony of a unique femur as abscesses were present in all ten sectioned femurs, five femurs per group. Scale bars at bottom right of each image represent 100 μm.
